# Age and Sex-Dependent Differences in the Neurochemical Characterization of Calcitonin Gene-Related Peptide-Like Immunoreactive (CGRP-LI) Nervous Structures in the Porcine Descending Colon

**DOI:** 10.3390/ijms20051024

**Published:** 2019-02-27

**Authors:** Krystyna Makowska, Slawomir Gonkowski

**Affiliations:** Department of Clinical Physiology, Faculty of Veterinary Medicine, University of Warmia and Mazury in Olsztyn, Oczapowski Str. 13, 10-718 Olsztyn, Poland; krystyna.makowska@uwm.edu.pl

**Keywords:** calcitonin gene-related peptide, enteric nervous system, aging, sex, porcine

## Abstract

Neurons of the enteric nervous system (ENS) may undergo changes during maturation and aging, but knowledge of physiological stimuli-dependent changes in the ENS is still fragmentary. On the other hand, the frequency of many ENS-related intestinal illnesses depends on age and/or sex. The double immunofluorescence technique was used to study the influence of both of these factors on calcitonin gene-related peptide (CGRP)—positive enteric nervous structures—in the descending colon in young and adult female and castrated male pigs. The influence of age and gender on the number and neurochemical characterization (i.e., co-localization of CGRP with substance P, nitric oxide synthase, galanin, cocaine- and amphetamine-regulated transcript peptide and vesicular acetylcholine transporter) of CGRP-positive nerve structures in the colonic wall has been shown. These observations strongly suggest the participation of CGRP in adaptive processes in the ENS during GI tract maturation. Moreover, although the castration of males may mask some aspects of sex-dependent influences on the ENS, the sex-specific differences in CGRP-positive nervous structures were mainly visible in adult animals. This may suggest that the distribution and exact role of this substance in the ENS depend on the sex hormones.

## 1. Introduction

The enteric nervous system (ENS) is responsible for the intrinsic innervation of the gastrointestinal (GI) tract. It is situated in the wall of the entire digestive tract, from the esophagus to the rectum. The enteric plexuses consist of millions of neurons, which play various roles and express a range of neurotransmitters and/or neuromodulators [1]. Because of the high level of autonomy in the activity of the enteric neurons, the ENS is considered to be the “second” or “intestinal brain” [2].

Depending on the animal species and the fragment of the GI tract, the ENS structure shows some differences. In the esophagus and stomach, the enteric neurons of large mammals (e.g., pigs) are grouped into two ganglionated plexuses: the myenteric plexus (MP)—located between longitudinal and circular muscle layers and the submucous plexus placed near the lamina propria of the mucosal layer. In the small and large intestines, the submucous plexus is additionally divided into the outer submucous plexus (OSP) and inner submucous plexus (ISP), which are placed in the submucosal layer: the OSP near the inner side of the circular muscle layer and the ISP near the lamina propria of the mucosa [3,4,5].

It should be emphasized that enteric neurons are very diverse in terms of morphological, functional and electrophysiological properties, but the most important criterion of neuronal classification in the ENS is the neurochemical characterization of nerve cells [3,4]. To date, several dozen neuronal active substances have been found in the enteric neurons. They may play various functions and are involved in all aspects of gastrointestinal physiology, such as motility, excretive activity, intestinal blood flow and ion transport [1,6]. Previous studies have shown that the same neuronal cells may contain even a few active substances, which usually play similar roles [7,8] and studies on the co-localization of these substances contribute to a better understanding of their functions.

It is also known that enteric neurons may undergo changes concerning their structural, functional and chemical phenotype as a result of adaptive and/or neuroprotective responses to various pathological and physiological agents [7,9,10,11,12,13,14], but knowledge of these issues is still fragmentary. In particular, little is known about the reactions of the enteric neurons to the maturation and aging of the organism [15] and many aspects of this problem are still completely unknown. The second area of knowledge about the ENS which still remains obscure concerns sex-dependent differences in its organization. Despite some suggestions that, in males and females, the enteric neurons contain various active substances and respond differently to pathological factors [16], exact studies on sex-dependent differences in the neurochemical characterization of neuronal cells in the ENS have not been conducted.

One neuronal substance which plays an important and multidirectional role in the ENS is calcitonin gene-related peptide (CGRP). Since the first identification of CGRP in 1982, the wide distribution of this peptide has been described in both the central and peripheral nervous systems [17,18]. In the GI tract of various mammal species, including humans, the presence of CGRP has been noted in all types of enteric plexuses from the esophagus to the rectum [11,19,20,21]. At first, CGRP is known as one of the most important factors participating in the conduction of sensory and pain stimuli [12]. However, other studies have reported that CGRP is also involved in the suppression of gastric acid secretion, the relaxation of the GI muscular membrane, vasodilatation processes and regulation of the absorption of nutrients from the intestines [22,23,24]. Moreover, it is also known that the expression of this neuropeptide in enteric nervous structures undergoes changes under the influence of some pathological stimuli [7,9], which suggests the participation of CGRP in adaptive and/or neuroprotective processes and the probable protective function of this substance against harmful factors [25]. Nevertheless, many aspects of the CGRP functions in the ENS are still not fully understood. The previous reports on the impact of physiological conditions, such as the development and maturation of the digestive tract on the population of CGRP-positive nervous structures are rather scarce and apply only to nerve fibers [10,26,27], and the influence of gender on these populations has not yet been studied.

Therefore, the aim of the present study was to investigate the impact of selected physiological states (such as age and gender) on the number, distribution and neurochemical characterization of CGRP-positive nervous structures in the wall of the porcine descending colon. Both the selection of domestic pig as an experimental animal and the descending colon as a fragment of the GI tract included in the study were not accidental. Due to the anatomical, neurochemical and electrophysiological similarities in the ENS organization between humans and pigs [28], this species is considered to be an optimal animal model (significantly better than using rodents) for studies on the influence of various stimuli on the ENS in humans. In turn, the descending colon is where numerous pathological states occur, and many of them are connected with the aging of the organism [26] and/or depend on gender [29]. The present study not only contributes to the elucidation of the exact roles of CGRP in the maturation of the GI tract in individuals of the opposite sex, but it may also have practical applications. Namely, it may be the first step to a better understanding of the roles of the ENS during disorders connected with age and gender, which may contribute to improving the treatment of various intestinal diseases in the future.

## 2. Results

### 2.1. The Number of CGRP-Positive Enteric Nervous Structures

**Figure 1:**
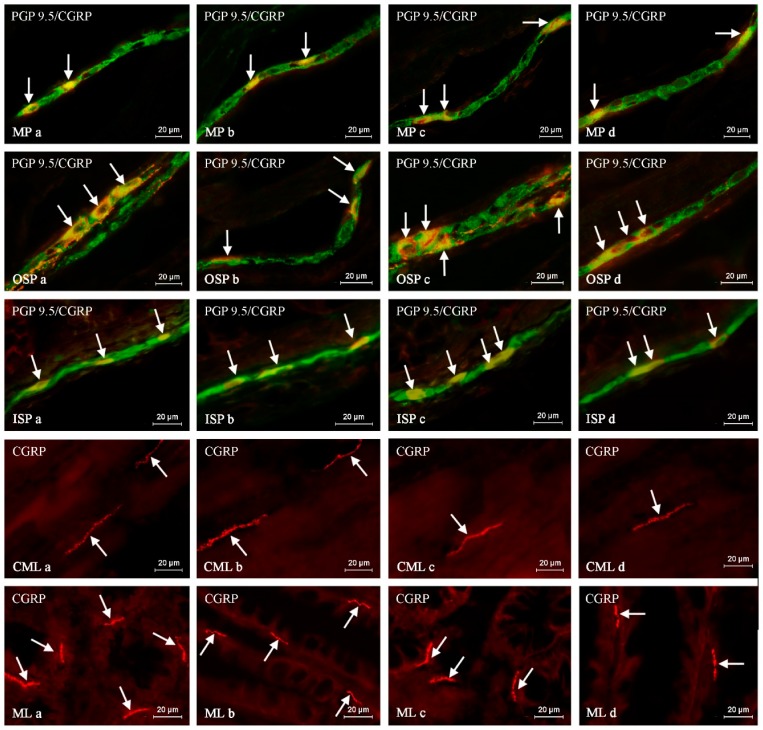
The representative images of the distribution of calcitonin gene-related peptide (CGRP) in the nervous structures of the myenteric plexus (MP), outer submucous plexus (OSP), inner submucous plexus (ISP), circular muscle (CML) and mucosal (ML) layers of the porcine descending colon under physiological conditions in young females (**a**), young males (**b**), adult females (**c**) and adults males (**d**). Images are composites of merged photographs taken separately from green (pan-neuronal marker PGP 9.5) and red (CGRP) fluorescent channels. Nervous structures immunoreactive to CGRP are indicated by arrows.

Throughout the study, CGRP-like immunoreactive (CGRP-LI) nervous structures were observed in the descending colon of all groups of animals studied (Table 1, Figure 1).

In young animals, the percentage of CGRP-LI enteric neurons and the density of intra-mucosal and intramuscular nerves immunoreactive to CGRP were similar in both females and males (Table 1). The number of CGRP-positive neurons was higher in both types of submucous plexuses than in the MP, and the density of such nerves was higher in the mucosal layer than in the muscles.

Contrary to young animals, for adult pigs, statistically significant differences in the number of CGRP-LI neurons in all types of enteric plexuses between males and females were observed. Contrary to enteric neurons, for CGRP-LI nerve fibers located in the muscular and mucosal layers, no statistically significant differences were observed between mature females and males.

Statistically significant differences were noted between young females and females who had undergone puberty. The percentage of CGRP-LI neurons in all types of the enteric plexuses was higher in the adult females and the number of nerve fibers in the mucosal and muscular layers was lower (Table 1). In turn, the amount of CGRP-positive neurons in all enteric plexuses in the males was similar in young and mature animals. However, the differences in the number of muscular and mucosal CGRP-LI nerve fibers (with a slight decrease) between these groups were statistically significant. The results concerning the percentage of CGRP-positive neuronal structures in the ENS of the descending colon are summarized in Table 1.

### 2.2. Co-Localization of CGRP and SP

**Figure 2:**
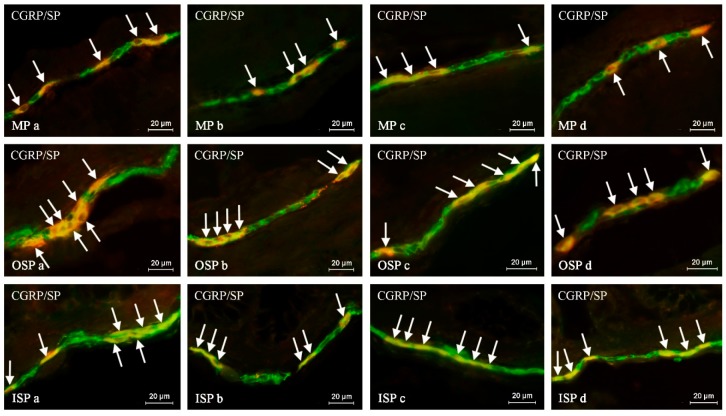
The representative images of the co-localization of calcitonin gene-related peptide (CGRP) with substance P (SP) in the neurons of the myenteric plexus (MP), outer submucous plexus (OSP) and inner submucous plexus (ISP) of the porcine descending colon under physiological conditions in young females (**a**), young males (**b**), adult females (**c**) and adults males (**d**). The images are composites of merged photographs taken separately from green (CGRP) and red (SP) fluorescent channels. Nervous structures, where CGRP co-localizes with SP, are indicated by arrows.

During the present study, the co-localization of CGRP with all substances studied was noted in the nervous structures within the descending colon of all animal groups. The degree of co-localization clearly depended on the type of neuronal factor studied and the part of the ENS. It was also found that the occurrence of the studied substances in the CGRP-LI nervous structures fluctuated depending on the sex and age of the animals. The largest percentage of neurons immunoreactive to CGRP also revealed the presence of SP (Table 2, Figure 2). Contrary to neurons, in the nerve fibers within the muscular and mucosal layers, the co-localization of these substances was significantly lower. In young males, the degree of co-localization of CGRP and SP in the enteric neurons was lower than in females (Table 2). In turn, the percentage of CGRP+/SP+ nerve fibers in the muscular and mucosal layers in young animals of both genders was similar.

In adult animals of both genders, the degree of co-localization of SP and CGRP was lower in all types of enteric plexuses and in intra-mucosal fibers in comparison to the young pigs (Table 2). In turn, the number of CGRP-LI fibers in the muscular layer in young and adult females and males was similar (Table 2).

Comparing adult animals of both sexes, a lower degree of co-localization of CGRP and SP in the MP and OSP was noted in males, while the values observed in other parts of the colonic wall did not show statistically significant differences between both sexes. Results concerning the co-localization of CGRP and SP are summarized in Table 2.

### 2.3. Co-Localization of CGRP and VAChT

Another substance showing a high degree of co-localization with CGRP within the enteric neuronal structures was VAChT—a marker of cholinergic neurons. In young animals of both genders, the degree of co-localization of CGRP and VAChT was similar for neurons located in the MP and ISP, as well as fibers placed in the muscular and mucosal layers (Table 3). Only the percentage of CGRP+/VAChT+ neurons in the OSP of young males was statistically significantly higher than in young females (Table 3).

The maturation of females did not cause statistically significant changes in the degree of co-localization of CGRP and VAChT (Table 3). Contrary to females, in adult males, a visible decrease (in comparison to young males) in the degree of co-localization of CGRP and VAChT in the neurons within the MP and ISP, as well as in the nerves located in the mucosal layer, was noted (Table 3). On the other hand, statistically significant differences in the co-localization of CGRP and VAChT between adult females and males were not observed. The results concerning the co-localization of CGRP and VAChT are summarized in Table 3.

### 2.4. Co-Localization of CGRP and CART

In the present study, it was noted that numerous CGRP-LI enteric neurons and nerve fibers in the colonic wall were also immunoreactive to CART. The degree of co-localization of these substances was similar in young animals of both sexes with the exception of the muscular layer, where the number of CGRP+/CART+ nerves was lower in males (Table 4).

The maturation of animals of both sexes caused an increase in the degree of co-localization of CGRP and CART in neurons of all types of the enteric plexuses (Table 4). On the other hand, the percentage of CGRP+/CART+ nerves (in relation to all CGRP-LI fibers) in mature females was not significantly statistically different from those noted in young sows. In males, the maturation caused an increase in the co-localisation of these substances in nerve fibers located in the muscular layer without changes in intra-mucosal nerves (Table 4). By comparing adult animals of both sexes, a higher degree of co-localization of CGRP and CART in the OSP was noted in males, while other values were similar. The results of the co-localization of CGRP and CART are summarized in Table 4.

### 2.5. Co-Localization of CGRP and nNOS

**Figure 3:**
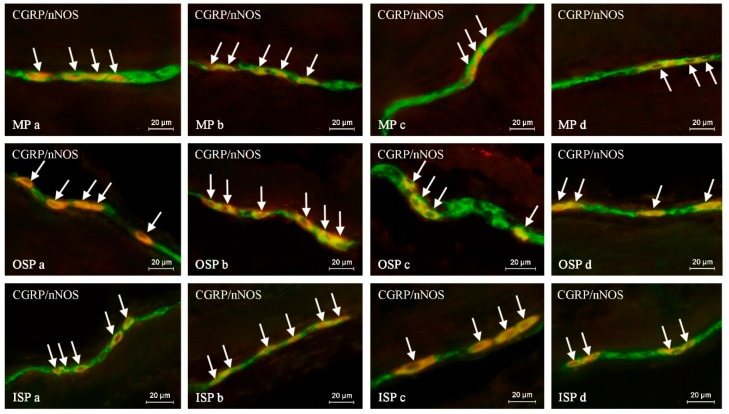
The representative images of the co-localization of calcitonin gene-related peptide (CGRP) with neuronal isoform of nitric oxide synthase (nNOS) in the neurons of the myenteric plexus (MP), outer submucous plexus (OSP) and inner submucous plexus (ISP) of the porcine descending colon under physiological conditions in young females (**a**), young males (**b**), adult females (**c**) and adults males (**d**). Images are composites of merged photographs taken separately from green (CGRP) and red (nNOS) fluorescent channels. Nervous structures where CGRP co-localizes with nNOS are indicated by arrows.

The degree of co-localization of CGRP with nNOS within the enteric neurons of the porcine descending colon was also relatively high, contrary to intra-mucosal and intramuscular nerves (Figure 3). In young males, the degree of co-localization of CGRP and nNOS in neurons within all types of enteric plexuses was higher than in females, while the percentage of CGRP+/nNOS+ nerves in the muscular and mucosal layers was similar in young animals of both sexes (Table 5).

In adult animals of both sexes, the percentage of CGRP+/nNOS+ neuronal cells in comparison to young pigs was lower in all types of enteric plexuses. Contrary to neuronal cells, the maturation of females did not affect the percentage of intra-mucosal and intramuscular CGRP+/nNOS+ nerves (Table 5). In males, the maturation caused a decrease in the number of intramuscular CGRP+/nNOS+ nerves without changes within the fibers located in the mucosal layer (Table 5). During the present study, statistically significant differences in the degree of co-localization of CGRP with nNOS within colonic nervous structures between adult animals of both sexes were not noted. The results concerning the co-localization of CGRP and nNOS are summarized in Table 5.

### 2.6. Co-Localization of CGRP and GAL

During the present study, the co-localization of CGRP with GAL in the nerve structures within the porcine descending colon was also noted. In young males, the degree of co-localization of CGRP and GAL in the enteric neurons, as well as in the intramuscular nerves, was slightly lower than in young females, while the number of CGRP+/GAL+ fibers in the mucosal layer was similar in young animals of both sexes (Table 6).

The maturation in females caused a statistically significant decrease in the number of GGRP+/GAL+ neurons in the OSP and ISP. The percentage of such neurons in the MP, as well as the number of CGRP+/GAL+ intramuscular and intra-mucosal nerves, was similar in young and adult females (Table 6). In males, maturation caused a clear decrease in the degree of co-localization of CGRP and GAL in neurons located within all types of the enteric plexuses without changes in the number of CGRP+/GAL+ nerves in the muscular and mucosal layers.

By comparing adult animals of both sexes, a lower degree of co-localization of CGRP and GAL in neurons located within the MP and ISP was noted in males, while other values were similar in pigs of both sexes. The results of the co-localization of CGRP and GAL are summarized in Table 6.

## 3. Discussion

Previous studies have reported that the ENS may undergo changes under physiological stimuli, but the existing knowledge relating to these issues is rather scarce. It is known that nerve structures in the GI tract may be influenced by one’s diet, environmental factors or the composition of the gut microbiota [30,31,32], but the main factors which may affect the ENS are maturation and aging of the organism [26,27]. Studies conducted on humans and rodents have shown that maturation and aging not only substantially affect the number and morphology of the enteric neurons, but may also change the synaptic profiles and expression of selected neuronal substances in the ENS, and the character of these changes depends on the mammal species, the segment of the GI tract and the type of active substance studied [15,33,34].

The results obtained during the current study have shown age-dependent differences in the size of the population of CGRP-positive enteric neuronal structures and their neurochemical characterization in the porcine descending colon. Because the study was performed on adults and not older animals, these changes probably result from the development and maturation of the GI tract. On the other hand, their exact mechanisms are still unclear.

Given that the ENS undergoes major developmental and adaptive modifications during the maturation of the organism, including changes in neuronal morphology and electrophysiological properties, as well as the organization of the enteric synapses [35], the changes in the population of CGRP-LI nervous structures, first of all, may be connected with adaptive activities of this peptide, which monitor the condition of the intestine and maintenance of gut homeostasis [36,37]. This is more likely since, on the one hand, previous studies have reported maturation-dependent changes in length, number and appearance of axons and dendrites of the enteric neurons [35]. However, CGRP participates in physiological neuronal development through its involvement in the differentiation of neurons and the promotion of dendrite formation [38,39].

The next mechanism of observed differences may be connected with the participation of CGRP in the regulation of blood vessel functions. It is commonly known that the growth and maturation of the body require considerable vascular remodeling [40]. In turn, CGRP is known as a factor that not only regulates vascular functions and blood flow in the intestine [41], but also as a substance taking part in vasculogenesis during the development and maturation of the body through its influence on the expression of the vascular endothelial growth factor (VEGFA) [42,43].

The possibility that that changes in the number of CGRP-LI colonic nervous structures may result from other causes cannot be excluded. Because CGRP is involved in sensory stimuli conduction [12], it may be connected with the maturation-dependent remodeling of the sensory neurons described in previous studies [18]. In turn, the participation of CGRP in the control of the intestinal motility [24] suggests that observed fluctuations result from developmental age-dependent changes in the muscles of the GI tract and in intestinal motility [35,44]. Moreover, it is known that clear changes in the enteric nervous structures may be caused by the influence of the intestinal microbiota [31,45], and the relatively well-known differences in the composition of gut flora in young and adult animals [46] might have contributed to the observed changes. Another cause of the fluctuations in the number of CGRP-LI nervous structures (which among others is also connected with gut microbiota) may be connected with immunological processes [45]. This is reinforced by the relatively well-known maturation-dependent remodeling of the immune system [47], close correlations between the ENS and immune cells [48], as well as the participation of CGRP in immunological reactions [49].

It should be underlined that the exact direct mechanism of differences in the number of CGRP-LI enteric nervous structures noted in this study is also unknown. It may have resulted from fluctuations in various stages of protein synthesis. Nevertheless, based on the present results, which show a decrease in the number of CGRP-LI colonic nerve fibers along with a slight increase in the percentage of CGRP-LI neurons in females and without changes in such neurons in males, the most probable reasons for the noted fluctuations seem to be development-dependent changes in the intra-neuronal transport [50,51].

The observations made during the present study have also shown that the maturation of the body affects not only the number of CGRP-LI colonic nervous structures, but also changes (in a way, even more substantially) their neurochemical characterization. The obtained results, showing the presence of all active substances studied in CGRP-positive enteric neurons, have confirmed the considerable differentiation of such neuronal cells that were known from previous studies [7]. Interestingly, neuronal factors noted in CGRP-LI neurons during the present investigation often play opposing roles, which could mean that the CGRP is engaged in various mechanisms within the GI tract. For example, acetylcholine is the main excitatory neuromediator within the ENS, while nitric oxide is considered to be one of the most important inhibitory factors within the GI tract [52].

It is most likely that the participation of substances, which co-localize with CGRP in the enteric neurons in homeostasis maintenance in the colon during the growth and maturation of the gastrointestinal tract [36,37], have given rise to changes noted in the present study. The present study has shown that the degree of co-localization of CGRP with the majority of substances studied in the colonic enteric neurons is lower in adult animals. This is in agreement with the previous observations that the expression of acetylcholine, nitric oxide, GAL and SP in various parts of the nervous system was lower in older individuals [15]. Moreover, the differences in the degree of co-localization of CGRP and other studied substances may result from the same causes and mechanisms as mentioned above for changes in CGRP-LI nervous structures.

The second aim of the present study was to investigate, for the first time, the sex-dependent differences in CGRP-LI enteric nervous structures in the descending colon. It should be underlined that the existing information about differences in the ENS organization (including number, phenotype and activity of the enteric nervous structures) between males and females is much more limited and fragmentary than knowledge about changes in the ENS with the maturation and aging of the body [37]. Moreover, previous studies concerning this issue are inconclusive. Some of them have reported that there are no diversities between male and female ENS in certain species [53], while others have shown such differences in reaction to some pathological factors and stress conditions [16,54].

Generally, during the present study, the percentage of CGRP-positive enteric neurons was similar in young animals of both sexes, while it was higher in the adult females than in males. It should be highlighted that the age of the adult animals was the age of sexual maturity, although the males were castrated at around one week of age, which was the cause of different levels of reproductive hormones in males and females before and after puberty. Thus, the differences observed during the present study in animals of opposite genders probably result from various levels of female hormones. It is all the more probable since it is known that female sex hormones may affect a wide range of functions of the GI tract via various types of estrogen receptors located among others on the enteric neurons in both males and females [55,56].

Primarily female sex hormones in the ENS stimulate enteric neurons to produce selected neuroactive substances and protect them against damage factors [57,58]. Moreover, these hormones may affect the intestinal motility, blood flow and functions of the mucosal layer [16,29,37]. It is also known that female sex hormones regulate somatic and visceral sensitivity, especially in response to different pathological stimuli [57], and some studies suggest that the estrogenic compounds regulating the release of CGRP (the major sensory factor) could, at least in part, provide a rational explanation for the gender differences in visceral pain sensitivity, which is higher in females [58]. It should be underlined that CGRP is involved in the majority of above-mentioned gastro-intestinal processes affected by female sex hormones, including intestinal motility, protective mechanisms and mesenteric blood flow [22,23,24], which may be at the heart of the observed changes.

The observations made in the present study of gender differences (similar to age-dependent changes) concerned not only the percentage of CGRP-LI nervous structures, but also their neurochemical characterization. These differences were more visible in adult animals and they are probably connected with the above-discussed influence of female sex hormones on the GI tract and the ENS. The changes in the degree of co-localization of CGRP with other substances may have resulted from the role of CGRP as a neuromodulator, influencing the secretion of other neuronal factors [52]. However, they may also be connected with the direct influence of aging and/or gender on the synthesis and distribution of studied the neuromediators.

## 4. Materials and Methods

The experiment was performed on tissues collected from 20 pigs slaughtered at a commercial slaughterhouse. Fragments of the descending colon (from the same place in all animals, located about 20 cm before the anus) were taken immediately after the death of the pigs using a typical commercial method (carbon dioxide) and bowel removal. Depending on the age and sex of the animals, samples were divided into four groups (five animals in each). Descending colons were collected from young pigs before puberty (about 10 weeks old), the compounded group A (samples from females) and group B (males). Group C contained tissues from females after puberty (7–8 months old) and group D from mature males (also 7-8 months old). Both young and mature males were castrated at the age of about 1 week. All procedures during this study were performed according to Act for the Protection of Animals for Scientific or Educational Purposes of 15 January 2015 (Official Gazette 2015, No. 266), applicable in the Republic of Poland. Based on this act, due to the fact that tissues were taken from commercially slaughtered animals, approval from the Bioethical Committee for the present study was not required.

Immediately after collection, 2-cm (approx.) colon fragments were fixed in a solution of 4% buffered paraformaldehyde (pH 7.4). After one hour, the solution was changed into a phosphate buffer (0.1 M, pH 7.4). For the next three days, tissues were stored at 4 °C with a daily exchange of the buffer. Afterward, the colon fragments were put into 18% phosphate-buffered sucrose and stored for three weeks. The tissues were then frozen at −22 °C and cut perpendicular to the colonic lumen into 14-µm-thick sections using a freezing microtome (Microm, HM 525, Walldorf, Germany).

Such prepared descending colon fragments were subjected to the routine double-labeling immunofluorescence method, as described previously by Majewski et al. 2002 [59].

In brief, this technique was carried out as follows. At room temperature (rt), frozen tissue fragments were dried for 45 minutes and incubated for one hour with a blocking solution (10% goat serum, 0.1% bovine serum albumin (BSA), 0.01% NaN3, Triton X-100, and thimerosal in PBS). Next, the colon fragments were incubated with a mixture of two primary antibodies raised in different species. Depending on the labeling, the primary antisera were directed towards: protein gene product 9.5 (PGP 9.5, used as a pan-neuronal marker), calcitonin gene-related peptide (CGRP), substance P (SP), neuronal form of nitric oxide synthase (nNOS, used as a marker of nitrergic neurons), galanin (GAL), cocaine- and amphetamine-regulated transcript (CART) and vesicular acetylcholine transporter (VAChT, used as a marker of cholinergic neurons). During the incubation process, the slices covered with the mixture of antibodies were left overnight at rt in a humid chamber. The next day, incubation was performed with a mixture of species-specific secondary antisera (conjugated to Alexa Fluor 488 or 546; 1 h, rt), which enabled the visualization of the primary antibodies connected with suitable antigens. Triple rinsing of the samples (15 min. each) after each stage of the described method was made up using PBS (pH 7.4). The specifications and working dilutions of primary and secondary antisera used during the present investigation are presented in Table 7.

During the present study, routine tests of antibody specificity (including pre-absorption, omission and replacement of primary antibodies by non-immune sera) were performed. These tests completely eliminated specific stainings. It should be pointed out that CGRP occurs in two forms expressed from two genes CALCA and CALCB. Although both antibodies used in the present study are directed against the same form of CGRP (α CGRP encoded by CALCA), they were obtained from different species. Therefore, a comparative study between two anti-CGRP antibodies used during the investigation was performed. This study confirmed that both antibodies against CGRP bind to the same colonic nervous structures in the same pattern and frequency.

The labeled samples were observed under an Olympus BX51 microscope equipped with epi-fluorescence and appropriate filters. Only neurons with clearly-visible nucleus were included in these studies.

The percentage of CGRP-like immunoreactive (LI) neurons was evaluated in relation to nerve cells labeled with PGP-9.5, considered as 100%. At least 500 PGP-9.5-positive neurons were counted and examined for the presence of CGRP in each animal studied, within the MP, OSP and ISP. Moreover, to determine the degree of co-localization of CGRP with other neuronal factors studied, at least 150 CGRP-positive cell bodies in particular types of enteric ganglia were examined for immunoreactivity to each of the other substances and, in this part of the study, cells immunoreactive to CGRP were considered 100%. The obtained results were pooled and presented as mean ± SEM. To prevent the double counting of neurons, the sections included in the study were located at least 150 µm apart.

The density of intra-ganglionic CGRP-LI nerve fibers was evaluated using an arbitrary scale. In this case, the absence of the studied fibers was marked with (−), single fibers with (+), a rare network of fibers with (++), a dense meshwork with (+++) and a very dense meshwork with (++++). In turn, the evaluation of CGRP-positive nerves in the muscular and mucosal layers of the descending colon was based on the counting all CGRP-positive fibers per observation field (0.55 mm^2^). Nerves were counted in four fragments of the descending colon located at least 150 µm apart per animal (in five observation fields per section) and the obtained data were pooled and presented as a mean. In turn, the denotation of the neurochemical characterization of CGRP-LI nerves in the colonic muscular and mucosal layers was based on the counting of at least 100 nerves immunoreactive to CGRP and by evaluating each of them for immunoreactivity to each of the other studied neuronal factors. The obtained data were also pooled and presented as the mean percentage ± SEM (CGRP-positive nerves were considered to represent 100%). To prevent the double counting of the nerve fibers, in all the above-mentioned methods, the evaluated sections of the colon were located at least 200 μm apart.

A statistical analysis was performed with Student’s *t*-test (Statistica 12, StatSoft, Inc., Cracow, Poland) and the differences were considered statistically significant at *p* < 0.05. To evaluate the sex-dependent differences in the ENS, the results obtained from males and females in each age group were compared. Moreover, the results were compared in young and mature animals of the same sex to investigate the effect of the development and maturation of the GI tract on the enteric nervous structures immunoreactive to CGRP.

## 5. Conclusions

To sum up, the obtained results clearly demonstrate that CGRP is widely distributed in the nervous structures in the descending colon of male and female pigs of different ages. Moreover, the present study, for the first time, describes differences in the number and neurochemical characterization of colonic CGRP-LI nervous structures between young and adult animals. These differences strongly suggest the participation of this peptide in adaptive and developmental processes within the digestive tract, as well as in mechanisms connected with body growth and maturation. The received findings also show that the level of sex hormones clearly affects the population of CGRP-LI neurons and colonic nerve fibers and the degree of co-localization of CGRP with other neuronal factors. Nevertheless, the elucidation of the exact mechanisms connected with the involvement of this substance in intra-intestinal physiological processes dependent on maturation and/or gender requires further study.

## Figures and Tables

**Table 1:** The calcitonin gene-related peptide (CGRP)-like immunoreactive (CGRP-LI) perikarya and nerve fibers in the porcine descending colon under physiological conditions in young (10-week old) females and males and adult (6–8 months old) females and males.

		Young Females	Young Males	Adult Females	Adult Males
CML ^1^		1.39 ± 0.086 ^a^	1.15 ± 0.095 ^d^	0.82 ± 0.107 ^a^	0.59 ± 0.050 ^d^
MP	CB ^2^	27.00 ± 0.256 ^a^	27.77 ± 1.035	29.46 ± 0.328 ^ac^	27.41 ± 0.337 ^c^
	NF ^3^	++	++	++	++
OSP	CB ^2^	38.85 ± 0.503 ^a^	38,01 ± 0,816	45.29 ± 0.802 ^ac^	38.09 ± 0.915 ^c^
	NF ^3^	++	++	++	++
ISP	CB ^2^	39.67 ± 0.365 ^a^	39.15 ± 1.655	44,84 ± 0.930 ^ac^	38.96 ± 1.912 ^c^
	NF ^3^	+	+	+	+
ML ^1^		5.05 ± 0.085 ^a^	4.71 ± 0.122 ^d^	3.75 ± 0.169 ^a^	3.45 ± 0.106 ^d^

CML: circular muscle layer; MP: myenteric plexus; OSP: outer submucous plexus; ISP: inner submucous plexus; ML: mucosal layer; CB: cell bodies; NF: nerve fibers. ^1^ The average number of nerve fibers per area studied (mean ± SEM). ^2^ The relative frequency of particular neuronal subclasses presented as a percentage (mean ± SEM) of all neurons counted within the ganglia stained for PGP 9.5 (used as a pan-neuronal marker). ^3^ The density of intra-ganglionic nerve fibers positive for CGRP is presented in arbitrary units_._ Statistically significant (*p* ≤ 0.05) differences are marked as follows: between young and adult females with ^a^, between young females and males with ^b^, between adult females and males with ^c^, between young and adult males with ^d.^

**Table 2:** The co-localization of CGRP with substance P (SP) in the enteric nervous structures of the porcine descending colon under physiological conditions in young (10-week old) females and males and adult (6–8 months old) females and males.

CGRP/SP		Young Females	Young Males	Adult Females	Adult Males
CML ^1^		22.87 ± 1.016	20.98 ± 0.537	20.23 ± 0.794	19.04 ± 0.804
MP	CB ^2^	55.60 ± 0.632 ^ab^	51.97 ± 0.883 ^bd^	52.96 ± 0.839 ^ac^	49.37 ± 0.490 ^cd^
OSP	CB ^2^	63.17 ± 0.635 ^ab^	55.75 ± 0.423 ^bd^	58.16 ± 1.759 ^ac^	51.68 ± 1.154 ^cd^
ISP	CB ^2^	64.28 ± 0.947 ^ab^	58.96 ± 1.90 ^b^	58.84 ± 1.346 ^a^	56.36 ± 1.288
ML ^1^		17.37 ± 0.625 ^a^	16.28 ± 0.425 ^d^	15.55 ± 0.188 ^ac^	14.00 ± 0.410 ^cd^

CML: circular muscle layer; MP: myenteric plexus; OSP: outer submucous plexus; ISP: inner submucous plexus; ML: mucosal layer; CB: cell bodies; NF: nerve fibers. ^1^ The average percentage of SP: positive nerve fibers with regard to all nerves immunoreactive to CGRP (mean ± SEM). ^2^ The relative frequency of SP: positive neurons presented as a percentage (mean ± SEM) of all neurons counted within the ganglia stained for CGRP. Statistically significant (*p* ≤ 0.05) differences are marked as follows: between young and adult females with ^a^, between young females and males with ^b^, between adult females and males with ^c^, between young and adult males with ^d^.

**Table 3:** The co-localization of CGRP with a vesicular acetylcholine transporter (VAChT) in the enteric nervous structures of the porcine descending colon under physiological conditions in young (10-week old) females and males and adult (6–8 months old) females and males.

CGRP/VAChT		Young Females	Young Males	Adult Females	Adult Males
CML ^1^		73.52 ± 0.968	72.40 ± 0.369	71.20 ± 0.583	71.11 ± 0.844
MP	CB ^2^	53.87 ± 1.506	57.35 ± 1.050 ^d^	49.01 ± 2.781	53.01 ± 0.856 ^d^
OSP	CB ^2^	55.00 ± 0.519 ^b^	59.21 ± 1.499 ^b^	53.19 ± 1.371	55.04 ± 1.383
ISP	CB ^2^	63.92 ± 1.780	64.57 ± 0.719 ^d^	58.55 ± 1.780	59.00 ± 1.767 ^d^
ML ^1^		74.51 ± 1.511	73.30 ± 0.871 ^d^	71.49 ± 0.500	70.48 ± 0.571 ^d^

CML: circular muscle layer; MP: myenteric plexus; OSP: outer submucous plexus; ISP: inner submucous plexus; ML: mucosal layer; CB: cell bodies; NF: nerve fibers. ^1^ The average percentage of VAChT: positive nerve fibers with regard to all nerves immunoreactive to CGRP (mean ± SEM). ^2^ The relative frequency of VAChT: positive neurons presented as a percentage (mean ± SEM) of all neurons counted within the ganglia stained for CGRP. Statistically significant (p ≤ 0.05) differences are marked as follows: between young and adult females with ^a^, between young females and males with ^b^, between adult females and males with ^c^, between young and adult males with ^d.^

**Table 4:** The co-localization of CGRP with cocaine- and amphetamine-related peptide (CART) in the enteric nervous structures of the porcine descending colon under physiological conditions in young (10-week old) females and males and adult (6–8 months old) females and males.

CGRP/CART		Young Females	Young Males	Adult Females	Adult Males
CML ^1^		65.62 ± 1.200 ^b^	60.57 ± 0.873 ^bd^	66.56 ± 1.874	66.48 ± 0.571 ^d^
MP	CB ^2^	40.82 ± 0.463 ^a^	40.28 ± 0.861 ^d^	42.82 ± 0.720 ^a^	42.96 ± 0.744 ^d^
OSP	CB ^2^	44.04 ± 0.748 ^a^	44.23 ± 1.356 ^d^	46.80 ± 0.907 ^ac^	49.77 ± 0.867 ^cd^
ISP	CB ^2^	59.56 ± 0.204 ^a^	55.59 ± 2.559 ^d^	61.25 ± 0.649 ^a^	62.00 ± 1.080 ^d^
ML ^1^		60.22 ± 0.916	59.27 ± 0.417	61.71 ± 0.579	61.17 ± 0.976

CML: circular muscle layer; MP: myenteric plexus; OSP: outer submucous plexus; ISP: inner submucous plexus; ML: mucosal layer; CB: cell bodies; NF: nerve fibers. ^1^ The average percentage of CART: positive nerve fibers with regard to all nerves immunoreactive to CGRP (mean ± SEM). ^2^ The relative frequency of CART-positive neurons presented as a percentage (mean ± SEM) of all neurons counted within the ganglia, stained for CGRP. Statistically significant (*p* ≤ 0.05) differences are marked as follows: between young and adult females with ^a^, between young females and males with ^b^, between adult females and males with ^c^, between young and adult males with ^d^.

**Table 5:** The co-localization of CGRP with a neuronal isoform of nitric oxide synthase (nNOS) in the enteric nervous structures of the porcine descending colon under physiological conditions in young (10-week old) females and males and adult (6-8 months old) females and males.

CGRP/nNOS		Young Females	Young Males	Adult Females	Adult Males
CML ^1^		2.53 ± 0.632	3.23 ± 0.374 ^d^	1.73 ± 0.461	1.96 ± 0.298 ^d^
MP	CB ^2^	38.58 ± 0.182 ^ab^	40.82 ± 0.953 ^bd^	37.29 ± 0.528 ^a^	38.37 ± 0.465 ^d^
OSP	CB ^2^	48.11 ± 0.427 ^ab^	51.30 ± 0.961 ^bd^	45.03 ± 1.257 ^a^	47.21 ± 1.052 ^d^
ISP	CB ^2^	58.08 ± 0.822 ^ab^	60.23 ± 0.386 ^bd^	52.03 ± 1.046 ^a^	54.19 ± 1.143 ^d^
ML ^1^		2.51 ± 0.226	2.35 ± 0.216	1.53 ± 0.473	1.38 ± 1.128

CML: circular muscle layer; MP: myenteric plexus; OSP: outer submucous plexus; ISP: inner submucous plexus; ML: mucosal layer; CB: cell bodies; NF: nerve fibers. ^1^ The average percentage of nNOS: positive nerve fibers with regard to all nerves immunoreactive to CGRP (mean ± SEM). ^2^ The relative frequency of nNOS: positive neurons presented as a percentage (mean ± SEM) of all neurons counted within the ganglia stained for CGRP. Statistically significant (*p* ≤ 0.05) differences are marked as follows: between young and adult females with ^a^, between young females and males with ^b^, between adult females and males with ^c^, between young and adult males with ^d^.

**Table 6:** The co-localization of CGRP with galanin (GAL) in the enteric nervous structures of the porcine descending colon under physiological conditions in young (10-week old) females and males and adult females and males (Group D).

CGRP/GAL		Young Females	Young Males	Adult Females	Adult Males
CML ^1^		38.12 ± 0.795 ^b^	35.80 ± 0.551 ^b^	36.24 ± 0.344	32.01 ± 2.078
MP	CB ^2^	38.90 ± 0.278 ^b^	37.28 ± 0.304 ^bd^	37.99 ± 0.246 ^c^	36.34 ± 0.268 ^cd^
OSP	CB ^2^	43.73 ± 0.569 ^ab^	42.24 ± 0.235 ^bd^	41.41 ± 0.371 ^a^	41.39 ± 0.248 ^d^
ISP	CB ^2^	52.35 ± 0.448 ^ab^	49.34 ± 0.881 ^bd^	49.56 ± 0.842 ^ac^	46.95 ± 0.445 ^cd^
ML ^1^		33.03 ± 1.910	30.54 ± 0.672	29.46 ± 0.850	28.87 ± 1.698

CML: circular muscle layer; MP: myenteric plexus; OSP: outer submucous plexus; ISP: inner submucous plexus; ML: mucosal layer; CB: cell bodies; NF: nerve fibers. ^1^ The average percentage of GAL: positive nerve fibers with regard to all nerves immunoreactive to CGRP (mean ± SEM) ^2^ The relative frequency of GAL: positive neurons presented as a percentage (mean ± SEM) of all neurons counted within the ganglia stained for CGRP. Statistically significant (*p* ≤ 0.05) differences are marked as follows: between young and adult females with ^a^, between young females and males with ^b^, between adult females and males with ^c^, between young and adult males with ^d^.

**Table 7:** The list of antisera and reagents used in immunohistochemical investigations.

**Primary Antibodies**				
**Antigen**	**Code**	**Species**	**Working Dilution**	**Supplier**
PGP 9.5	7863-2004	Mouse	1:1000	BioRad, Hercules, CA, USA
CGRP	T-5027	Guinea Pig	1:1600	BMA Biomedicals, Augst, Switzerland
CGRP	AB5920	Rabbit	1:1600	Millipore, Temecula, CA, USA
SP	8450-0505	Rat	1:1000	BioRad
nNOS	AB5380	Rabbit	1:2000	Sigma-Aldrich, Saint Louis, MO, USA
GAL	T-5036	Guinea Pig	1:2000	Peninsula Labs, San Carlos, CA, USA
CART	H-003-61	Rabbit	1:8000	Phoenix Pharmaceuticals, Inc., Burlingame, CA, USA
VAChT	H-V006	Rabbit	1:2000	Phoenix Pharmaceuticals
**Secondary Antibodies**				
**Reagents**			**Working Dilution**	**Supplier**
Alexa Fluor 488 donkey anti-mouse IgG			1:1000	ThermoFisher Scientific, Waltham, MA, USA
Alexa Fluor 488 donkey anti-rabbit IgG			1:1000	ThermoFisher Scientific
Alexa Fluor 488 donkey anti-guinea pig IgG			1:1000	ThermoFisher Scientific
Alexa Fluor 546 donkey anti-mouse IgG			1:1000	ThermoFisher Scientific
Alexa Fluor 546 donkey anti-rabbit IgG			1:1000	ThermoFisher Scientific
Alexa Fluor 546 donkey anti-rat IgG			1:1000	ThermoFisher Scientific
Alexa Fluor 546 donkey anti-guinea pig IgG			1:1000	ThermoFisher Scientific

## References

[1] Furness J.B., Callaghan B.P., Rivera L.R., Cho H.J. (2014). The enteric nervous system and gastrointestinal innervation: Integrated local and central control. Adv. Exp. Med. Biol..

[2] Schneider S., Wright C.M., Heuckeroth R.O. (2019). Unexpected roles for the second brain: Enteric nervous system as master regulator of bowel function. Annu. Rev. Physiol..

[3] Balemba O.B., Grøndahl M.L., Mbassa G.K., Semuguruka W.D., Hay-Smith A., Skadhauge E., Dantzer V. (1998). The organisation of the enteric nervous system in the submucous and mucous layers of the small intestine of the pig studied by VIP and neurofilament protein immunohistochemistry. J. Anat..

[4] Petto C., Gäbel G., Pfannkuche H. (2015). Architecture and chemical coding of the inner and outer submucous plexus in the colon of piglets. PLoS ONE.

[5] Balemba O.B., Mbassa G.K., Semuguruka W.D., Assey R.J., Kahwa C.K., Hay-Schmidt A., Dantzer V. (1999). The topography, architecture and structure of the enteric nervous system in the jejunum and ileum of cattle. J. Anat..

[6] Timmermans J.P., Adriaensen D., Cornelissen W., Scheuermann D.W. (1997). Structural organization and neuropeptidedistribution in the mammalian enteric nervous system, with special attention to those components involved in mucosal reflexes. Comp. Biochem. Physiol. A Physiol..

[7] Makowska K., Gonkowski S. (2018). The Influence of Inflammation and Nerve Damage on the Neurochemical Characterization of Calcitonin Gene-Related Peptide-Like Immunoreactive (CGRP-LI) Neurons in the Enteric Nervous System of the Porcine Descending Colon. Int. J. Mol. Sci..

[8] Wilhelm M., Lawrence J.J., Gábriel R. (2015). Enteric plexuses of two choline-acetyltransferase transgenic mouse lines: Chemical neuroanatomy of the fluorescent protein-expressing nerve cells. Brain Res. Bull..

[9] Vasina V., Barbara G., Talamonti L., Stanghellini V., Corinaldesi R., Tonini M., de Ponti F., de Giorgio R. (2006). Enteric neuroplasticity evoked by inflammation. Auton. Neurosci..

[10] Phillips R.J., Powley T.L. (2007). Innervation of the gastrointestinal tract: Patterns of aging. Auton. Neurosci..

[11] di Giancamillo A., Vitari F., Bosi G., Savoini G., Domeneghini C. (2010). The chemical code of porcine enteric neurons and the number of enteric glial cells are altered by dietary probiotics. Neurogastroenterol. Motil..

[12] Wolf M., Schrödl F., Neuhuber W., Brehmer A. (2007). Calcitonin gene-related peptide: A marker for putative primary afferent neurons in the pig small intestinal myenteric plexus?. Anat. Rec. (Hoboken).

[13] Samarasena J.B., Ahluwalia A., Tarnawski A.S., Shinoura S., Choi K.D., Lee J.G., Chang K.J. (2015). Expression of nerve growth factor, its TrkA receptor, and several neuropeptides in porcine esophagus. Implications for interactions between neural, vascular and epithelial components of the esophagus. J. Physiol. Pharmacol..

[14] Jungbauer C., Lindig T.M., Schrödl F., Neuhuber W., Brehmer A. (2006). Chemical coding of myenteric neurons with different axonal projection patterns in the porcine ileum. J. Anat..

[15] Saffrey M.J. (2013). Cellular changes in the enteric nervous system during ageing. Dev. Biol..

[16] Freire A.C., Basit A.W., Choudhary R., Piong C.W., Merchant H.A. (2011). Does sex matter? The influence of gender on gastrointestinal physiology and drug delivery. Int. J. Pharm..

[17] Warfvinge K., Edvinsson L. (2017). Distribution of CGRP and CGRP receptor components in the rat brain. Cephalalgia.

[18] Gangula P.R., Chauhan M., Reed L., Yallampalli C. (2009). Age-related changes in dorsal root ganglia, circulating and vascular calcitonin gene-related peptide (CGRP) concentrations in female rats: Effect of female sex steroid hormones. Neurosci. Lett..

[19] Makowska K., Obremski K., Zielonka L., Gonkowski S. (2017). The Influence of Low Doses of Zearalenone and T-2 Toxin on Calcitonin Gene Related Peptide-Like Immunoreactive (CGRP-LI) Neurons in the ENS of the Porcine Descending Colon. Toxins.

[20] Sundler F., Ekblad E., Håkanson R. (1991). Occurrence and distribution of substance P- and CGRP-containing nerve fibers in gastric mucosa: Species differences. Adv. Exp. Med. Biol..

[21] Wojtkiewicz J., Rytel L., Makowska K., Gonkowski S. (2017). Co-localization of zinc transporter 3 (ZnT3) with sensory neuromediators and/or neuromodulators in the enteric nervous system of the porcine esophagus. Biometals.

[22] Helton W.S., Mulholland M.M., Bunnett N.W., Debas H.T. (1989). Inhibition of gastric and pancreatic secretion in dogs by CGRP: Role of somatostatin. Am. J. Physiol..

[23] Barada K.A., Saadé N.E., Atweh S.F., Khoury C.I., Nassar C.F. (2000). Calcitonin gene-related peptide regulates amino acid absorption across rat jejunum. Regul. Pept..

[24] Katsoulis S., Conlon J.M. (1989). Calcitonin gene-related peptides relax guinea pig and rat gastric smooth muscle. Eur. J. Pharmacol..

[25] Abushik P.A., Bart G., Korhonen P., Leinonen H., Giniatullina R., Sibarov D.A., Levonen A.L., Malm T., Antonov S.M., Giniatullin R. (2017). Pro-nociceptive migraine mediator CGRP provides neuroprotection of sensory, cortical and cerebellar neurons via multi-kinase signaling. Cephalalgia.

[26] Phillips R.J., Pairitz J.C., Powley T.L. (2007). Age-related neuronal loss in the submucosal plexus of the colon of Fischer 344 rats. Neurobiol. Aging.

[27] Wang C., Houghton M.J., Gamage P.P., Collins H.E., Patel B.A., Yeoman M.S., Ranson R.N., Saffrey M.J. (2013). Changes in the innervation of the mouse internal anal sphincter during aging. Neurogastroenterol. Motil..

[28] Verma N., Rettenmeier A.W., Schmitz-Spanke S. (2011). Recent advances in the use of Sus scrofa (pig) as a model system for proteomic studies. Proteomics.

[29] Prusator D.K., Chang L. (2017). Sex-Related Differences in GI Disorders. Handb. Exp. Pharmacol..

[30] Giaroni C., De Ponti F., Cosentino M., Lecchini S., Frigo G. (1999). Plasticity in the enteric nervous system. Gastroenterology.

[31] Heiss C.N., Olofsson L.E. (2019). The role of the gut microbiota in development, function and disorders of the central nervous system and the enteric nervous system. J. Neuroendocrinol..

[32] Di Giancamillo A., Rossi R., Martino P.A., Aidos L., Maghin F., Domeneghini C., Corino C. (2018). Copper sulphate forms in piglet diets: Microbiota, intestinal morphology and enteric nervous system glial cells. Anim. Sci. J..

[33] Abalo R., Vera G., Rivera A.J., Martín M.I. (2007). Age-related changes in the gastrointestinal tract: A functional and immunohistochemical study in guinea-pig ileum. Life Sci..

[34] Bernard C.E., Gibbons S.J., Gomez-Pinilla P.J., Lurken M.S., Schmalz P.F., Roeder J.L., Linden D., Cima R.R., Dozois E.J., Larson D.W. (2009). Effect of age on the enteric nervous system of the human colon. Neurogastroenterol. Motil..

[35] Foong J.P. (2016). Postnatal development of the mouse enteric nervous system. Adv. Exp. Med. Biol..

[36] Cooke H.J. (1989). Role of the “little brain” in the gut in water and electrolyte homeostasis. FASEB J..

[37] Million M., Larauche M. (2016). Stress, sex and enteric nervous system. Neurogastroenterol. Motil..

[38] Harigai Y., Natsume M., Li F., Ohtani A., Senzaki K., Shiga T. (2011). Differential roles of calcitonin family peptides in the dendrite formation and spinogenesis of the cerebral cortex in vitro. Neuropeptides.

[39] D’Antoni S., Zambusi L., Codazzi F., Zacchetti D., Grohovaz F., Provini L., Catania M.V., Morara S. (2010). Calcitonin gene-related peptide (CGRP) stimulates purkinje cell dendrite growth in culture. Neurochem. Res..

[40] Unthank J.L., Lash J.M., Bohlen H.G. (1990). Maturation of the rat intestinal microvasculature from juvenile to early adult life. Am. J. Physiol..

[41] Nuki C., Kawasaki H., Kitamura K., Takenaga M., Kangawa K., Eto T., Wada A. (1993). Vasodilator effect of an adrenomeullin and calcitonin gene-related peptide receptors in rat mesenteric vascular beds. Biochem. Biophys. Res. Commun..

[42] Zheng S., Li W., Xu M., Bai X., Zhou Z., Han J., Shyy J.Y., Wang X. (2010). Calcitonin gene-related peptide promotes angiogenesis via AMP-activated protein kinase. Am. J. Physiol. Cell. Physiol..

[43] Yu X.J., Li C.Y., Wang K.Y., Dai H.Y. (2006). Calcitonin gene-related peptide regulates the expression of vascular endothelial growth factor in human HaCaT keratinocytes by activation of ERK1/2 MAPK. Regul. Pept..

[44] Maharaj A.R., Edginton A.N. (2016). Examining small intestinal transit time as a function of age: Is there evidence to support age-dependent differences among children?. Drug Metab. Dispos..

[45] Obata Y., Pachnis V. (2016). The Effect of Microbiota and the Immune System on the Development and Organization of the Enteric Nervous System. Gastroenterology.

[46] Singh P., Manning S.D. (2016). Impact of age and sex on the composition and abundance of the intestinal microbiota in individuals with and without enteric infections. Ann. Epidemiol..

[47] Stokes C.R. (2017). The development and role of microbial-host interactions in gut mucosal immune development. J. Anim. Sci. Biotechnol..

[48] Yoo B.B., Mazmanian S.K. (2017). The enteric network: Interactions between the immune and nervous systems of the gut. Immunity.

[49] de Jong P.R., Takahashi N., Peiris M., Bertin S., Lee J., Gareau M.G., Paniagua A., Harris A.R., Herdman D.S., Corr M. (2015). TRPM8 on mucosal sensory nerves regulates colitogenic responses by innate immune cells via CGRP. Mucosal Immunol..

[50] Moutaux E., Christaller W., Scaramuzzino C., Genoux A., Charlot B., Cazorla M., Saudou F. (2018). Neuronal network maturation differently affects secretory vesicles and mitochondria transport in axons. Sci. Rep..

[51] Fernandez H.L., Hodges-Savola C.A. (1994). Axoplasmic transport of calcitonin gene-related peptide in rat peripheral nerve as a function of age. Neurochem. Res..

[52] Aulí M., Martínez E., Gallego D., Opazo A., Espín F., Martí-Gallostra M., Jiménez M., Clavé P. (2008). Effects of excitatory and inhibitory neurotransmission on motor patterns of human sigmoid colon in vitro. Br. J. Pharmacol..

[53] Korolkiewicz R., Sliwinski W., Rekowski P., Halama A., Mucha P., Szczurowicz A., Guzowski P., Korolkiewicz K.Z. (1996). Contractile action of galanin analogues on rat isolated gastric fundus strips is modified by tachyphylaxis to substance P. Pharmacol. Res..

[54] Medland J.E., Pohl C.S., Edwards L.L., Frandsen S., Bagley K., Li Y., Moeser A.J. (2016). Early life adversity in piglets induces long-term upregulation of the enteric cholinergic nervous system and heightened, sex-specific secretomotor neuron responses. Neurogastroenterol. Motil..

[55] Liu J.Y.H., Lin G., Fang M., Rudd J.A. (2019). Localization of estrogen receptor ERα, ERβ and GPR30 on myenteric neurons of the gastrointestinal tract and their role in motility. Gen. Comp. Endocrinol..

[56] Zielinska M., Fichna J., Bashashati M., Habibi S., Sibaev A., Timmermans J.P., Storr M. (2017). G protein-coupled estrogen receptor and estrogen receptor ligands regulate colonic motility and visceral pain. Neurogastroenterol. Motil..

[57] Nie X., Xie R., Tuo B. (2018). Effects of Estrogen on the Gastrointestinal Tract. Dig. Dis. Sci..

[58] Pota V., Quagliariello V., Armenia E., Aurilio C., Passavanti M.B., Sansone P., Iannotti M., Catauro M., Coaccioli S., Barbarisi M. (2017). CGRP and Visceral Pain: The Role of Sex Hormones in In Vitro Experiment. J. Cell. Biochem..

[59] Majewski M., Kaleczyc J., Wasowicz K., Bossowska A., Gonkowski S., Klimaschewski L. (2002). Characterization of afferent and efferent galanin-containing nerve fibres in the porcine ovary. Folia Histochem. Cytobiol..

